# Cryopreservation of human colorectal carcinomas prior to xenografting

**DOI:** 10.1186/1471-2407-10-362

**Published:** 2010-07-08

**Authors:** Michael Linnebacher, Claudia Maletzki, Christiane Ostwald, Ulrike Klier, Mathias Krohn, Ernst Klar, Friedrich Prall

**Affiliations:** 1 Department of General Surgery, Division of Molecular Oncology and Immunotherapy, Clinic for Surgery, Schillingallee 35, 18057 Rostock, Germany; 2 Institute of Pathology; Strempelstr. 14, 18055 Rostock, University of Rostock, Rostock, Germany

## Abstract

**Background:**

Molecular heterogeneity of colorectal carcinoma (CRC) is well recognized, forming the rationale for molecular tests required before administration of some of the novel targeted therapies that now are rapidly entering the clinics. For clinical research at least, but possibly even for future individualized tumor treatment on a routine basis, propagation of patients' CRC tissue may be highly desirable for detailed molecular, biochemical or functional analyses. However, complex logistics requiring close liaison between surgery, pathology, laboratory researchers and animal care facilities are a major drawback in this. We here describe and evaluate a very simple cryopreservation procedure for colorectal carcinoma tissue prior to xenografting that will considerably reduce this logistic complexity.

**Methods:**

Fourty-eight CRC collected ad hoc were xenografted subcutaneously into immunodeficient mice either fresh from surgery (N = 23) or after cryopreservation (N = 31; up to 643 days).

**Results:**

Take rates after cryopreservation were satisfactory (71%) though somewhat lower than with tumor tissues fresh from surgery (74%), but this difference was not statistically significant. Re-transplantation of cryopreserved established xenografts (N = 11) was always successful. Of note, in this series, all of the major molecular types of CRC were xenografted successfully, even after cryopreservation.

**Conclusions:**

Our procedure facilitates collection, long-time storage and propagation of clinical CRC specimens (even from different centres) for (pre)clinical studies of novel therapies or for basic research.

## Background

The last decade has witnessed a tremendous progress in understanding the molecular pathology and the pathogenesis of colorectal carcinoma (CRC). Chromosomal as well as microsatellite instability and the CpG island methylator phenotype have been defined as major molecular pathogenetic mechanisms, giving rise to the main molecular classes of CRC [1,2]; and genome wide mutational analysis have shown that per individual cancer a limited number of signal transduction pathways are dysregulated by "driver" mutations in some (typically about 15) from a set of about 80 so-called candidate cancer genes [3,4].

In the wake of this, targeted therapies for CRC are beginning to enter the clinics, EGF-receptor blockade (with the pre-requisite of K-Ras mutational analysis) being the first already to be used more generally among these [5,6]. It may be expected for the near future that patient's tumor tissues, besides being subject to traditional histopathological examination, will be used for various additional molecular testings. While some of these can be done with conventional paraffin-embedded material, some will require frozen tumor tissue. But, conceivably, more elaborate molecular analyses or even functional tests eventually may be desirable, and for these analyses at least xenograft tumors may be prime choice [7-9].

However, xenografting as a routine will pose considerable logistical difficulties as technical expertise of different fields (surgery, pathology, molecular biology and animal care) must be brought together. Clearly, separating location and occasion when the tumor specimen accrues, the molecular analyses are done, and the engraftings are performed would rigorously reduce this logistical complexity. In addition, at least for research purposes, it would allow preselection of tumor specimens with desired molecular features in advance of the technically demanding xenografting procedures.

We here report an easy and effective method to store CRC tissue by cryopreservation for use in xenografting at a later date. Specifically, we aimed to explore feasibility and success rate in a consecutive series of CRCs collected ad hoc, comparing xenografting of tumor tissue fresh from surgery with xenografting after cryopreservation. In addition, we demonstrate that cryopreservation of established xenograft tumors for re-xenografting is also feasible. And finally, we show that a balanced distribution of the different molecular classes of CRCs will be obtained.

## Methods

### Tumor specimen collection and cryopreservation

Resection specimens of primary tumors (N = 48; primary CRCs without previous chemo-or radiotherapy) were received fresh from surgery. Tumor tissue cubes (ca. 3 × 3 × 3 mm) were cut from the deep invasive parts with a sterile scalpel blade.

Mirror blocks for cryostat sections were prepared from the adjacent parts of the tumours. Alternatively, xenograft tumors were removed under sterile conditions and pieces were taken from the peripheral parts of the tumors. Again, adjacent tumour tissues were used for cryostat sections. Typically, 4 tumor pieces were transferred into sterile cryo-tubes (greiner-bio-one, Frickenhausen, Germany) in 1.5 ml freezing medium (foetal calf serum containing 10% DMSO), sealed in a Freezing Container (Nalgene, Rochester, NY, USA), and placed immediately at -80°C. Until transplantation tubes were kept at -80°C (for a maximum of 6 weeks) or, after overnight cooling, transferred into liquid nitrogen (for longer storage periods). For xenografting, cryopreserved tumor pieces were thawed at 37°C. Prior informed consent was obtained in written from all patients, and all procedures were approved by the Ethics Committee of the University of Rostock (reference number II HV 43/2004) in accordance with generally accepted guidelines for the use of human material.

### Tumor xenografting

Tumor xenograftings were done by one of the following approaches: (I) xenografting of primaries on the day of surgery (n = 23); (II) xenografting of primaries after cryopreservation (n = 31); and (III) re-transplantation of xenografts after cryopreservation (n = 11). Tumor pieces were implanted subcutaneously uni-or bilaterally into the flanks of six to eight week old female mice under ether anaesthesia for a short period of time. We used NOD-SCID (NOD.CB17-Prkdcscid/J in cases of HROC24 to HROC46 and nude mice (NMRI nu/nu) for the subsequent xenograftings. As assessed by cryostat sections from the mirror blocks, there was seen an area fraction of 30 - 80% of viable tumour in the tissues used for xenografting. The numbers of mice receiving grafts and the numbers of grafts for each animal are given for each individual tumor in Table 1. Mice were kept in the animal facilities of the medical faculty of the University of Rostock and maintained in specified pathogen-free conditions. Animals were exposed to 12-h light/12-h darkness cycles and standard food and water including antibiotics (Co-trimoxazol) ad libitum. Their care and housing were in accordance with guidelines as put forth by the German Ethical Committee and the Guide for the Care and Use of Laboratory Animals (Institute of Laboratory Animal Resources, National Research Council; NIH Guide, vol.25, no.28, 1996). Growth of tumours to volumes of 1 - 1.5 cm^3 ^was taken as evidence of successful xenografting, and the animals were then sacrificed for collection of tumour tissues for further studies.

**Table 1:** Details of xenograftings and outcomes.

Tumor-ID	Fresh	Cryopreserved	Days frozen
HROC24	M1(+); M2(-); M3*		

HROC26	M1(-); M2(-); M3(-)		

HROC29	M1(+); M2(+); M3(-)		

HROC32	M2(+); M1*		

HROC33	M1(-); M2(-); M3(-)		

HROC37	M1(-); M2(-)		

HROC38	M1(-); M2(-)		

HROC39	M1(+); M2(+)		

HROC40	M1(+); M2(+)		

HROC45	M1(-); M2(-)		

HROC46	M1(+); M2(+)		

HROC48		M1(-;-); M2(-;-);M3(+;+); M4(+;-)	161/643

HROC50		M1(+;+); M2(-;+)	120

HROC51		M1(-;-); M2(-;-)	117

HROC52		M1(-;-); M2(-;-)	94

HROC53		M1(-;+); M2(-;-)	84

HROC54		M1(-;-); M2(-;+)	64

HROC55		M1(-;-); M2(-;-);M3(-;+); M4(-;-)	6/489

HROC56	M1(-); M2(-)		

HROC57	M1(+); M2(-)		

HROC59	M1(+;+); M2(-;+)		

HROC60	M1(-;+); M2(-;-)		

HROC61		M1(-;-); M2(-;-)	58

HROC62		M1(-;-); M2(+;-)	51

HROC63	M1(-;+); M2(-;-)	M1(-;-); M2(-;-)	88

HROC64		M1(+;-); M2(-;-)	78

HROC65	M1(+;-); M2(-;-)	M1(+;-); M2(-;-)	13

HROC66		M1(-;-); M2(-;-)	47

HROC67		M1(-;-); M2(-;-)	42

HROC68	M1(+;+); M2(+;+)	M1(-;+); M2(-;+)	38

HROC69	M1(+;+); M2(+;+)	M1(-;-); M2(-;-)	28

HROC70	M1(-;-); M2(-;+)	M1(-;+); M2(-;-)	27

HROC71		M1(+;+); M2(-;+)	27

HROC72		M1(+;+); M2(-;-)	240

HORC73		M1(-;-); M2(-;-)	238

HROC74		M1(+;+); M2(-;-)	230

HROC75		M1(-;+); M2(+;+)	223

HROC78		M1(+;+); M2(-;-)	188

HROC80		M1(-;+); M2(-;-)	119

HROC81		M1(-;-); M2(-;+)	118

HROC82		M1(+;+); M2(-;-)	118

HROC83		M1(-;-); M2(-;-)	87

HROC84		M1(-;+); M2(-;-)	73

HROC85	M1(-;+); M2(+;+)		

HROC86	M1(-;+)		

HROC87		M1(-;+); M2(-;+)	9

HROC88		M1(+;+); M2(-;+)	7

HROC89	M1(-;-); M2(+;-)	M1(+;+)	103

M denotes mice xenografted, the outcome is given in parentheses as index + or index -. One index is given for uni-and two indices for bilateral xenografts. * animals died due to problems with anaesthesia. Underlined: repetition of the xenografting procedure.

### Morphological and molecular studies

Dissections and histopathological examination of the primary tumors were done according to standard protocols for surgical pathology reports of CRCs [10], and additional staging information was compiled from patients' clinical charts. Primary tumors and xenografts were embedded in paraffin, and 4 μm H&E sections were obtained as well as β-catenin and MLH1 and MSH2 immunostainings.

The molecular analyses were done as previously published in detail [2]. Briefly, the Bethesda panel of microsatellite markers was used to test for microsatellite instability, chromosomal instability was assessed by DNA-flow cytometry and LOH analyses with various dinucleotide markers (D5S1385, D5S346 (5q21); D8S1734, D8S1771, NEFL (8p21); D9S942, D9S1748 (9p21); D17S1832, D17S250 (17p23); D18S70 (18q23)), and mutation analyses of the APC gene as well as the K-Ras and B-Raf genes were done. Finally, DNA-methylation was assessed by the MethyLight technology with the marker panel originally published by Ogino et al. and modified by us [2]. Based on these molecular data, tumors in this series were classified as sporadic standard type CRC (spStdCRC), sporadic high-degree microsatellite instable CRC (spMSI-H), hereditary non-polyposis CRC-type (HNPCC-type), or CpG island methylator phenotype CRC (CIMP-type).

### Verification of human origin of the xenograft tumors

A human specific PCR was performed by amplification of a portion of the human mitochondrial cytochrome b gene as previously described [11]. Briefly, the reaction mixture (25 μl) contained 25 ng of gDNA, 0.1 mM of each primer (L15674: TAGCAATAATCCCCATCCTCCATATAT, H15782: ACTTGTCCAATGATGGTAAAAGG), 200 μM dNTPs, 1 × standard reaction buffer and 0.1 U Taq DNA polymerase (Bioron, Ludwigshafen, Germany). PCR was performed in a standard thermal cycler for 40 cycles of 30 s at 96°C, 40 s at 59°C, and 1 min at 72°C. Products were separated on a 1% agarose gel and results were scored positive with the appearance of a band of 157 bp.

### Statistics

All data were entered into a computerized data bank (Statistical Package for the Social Sciences, SPSS version 13.0). Testing for significance of cross-tabulated data was done by two-sided Fisher's exact T-test. The criterion for significance was taken to be p < 0.05.

## Results

Overall, 48 primary CRCs were collected for these xenografting experiments. 47 were adenocarcinomas, and 1 tumor was a large cell neuroendocrine carcinoma (HROC 57). Information on histological and molecular types as well as staging information and patient characteristics are summarized in Table 2.

**Table 2:** Data of colorectal carcinomas used for xenografting experiments and overall results of outcomes.

Tumor-ID	Age/Gender	Site*	TNM-Stage	Molecular type^†^	Fresh	Cryo
HROC24	98/m	Right colon	G2T2N0M0	spMSI	Success	ND^‡^

HROC26	60/m	Left colon	G3T4N2M1	spStd	Failure	ND

HROC29	59/m	Right colon	G3T3N2M1	HNPCC	Success	ND

HROC32	82/f	Right colon	G2T4N2M1	spStd	Success	ND

HROC33	70/f	Left colon	G2T3N1M0	spStd	Failure	ND

HROC37	77/m	Right colon	G3T2N0M0	ND	Failure	ND

HROC38	67/f	Right colon	G2T3N0M0	spStd	Failure	ND

HROC39	69/m	Right colon	G3T4N0M0	spStd	Success	ND

HROC40	69/m	Left colon	G3T3N1M0	CIMP-H	Success	ND

HROC45	52/m	Right colon	G3T4N0M0	ND	Failure	ND

HROC46	66/m	Right colon	G3T3N0M1	spStd	Success	ND

HROC48	68/m	Right colon	G3T2N1M0	spMSI	ND	Success

HROC50	67/f	Right colon	G2T4N0M0	spMSI	ND	Success

HROC51	70/m	Left colon	G3T4N2M1	spStd	ND	Failure

HROC52	55/f	Left colon	G2T2N0M0	spStd	ND	Failure

HROC53	72/f	Right colon	G3T3N0M0	spMSI	ND	Success

HROC54	63/f	Left colon	G2T3N2M0	spStd	ND	Success

HROC55	81/f	Right colon	G3T2N0M0	spMSI	ND	Success

HROC56	70/m	Right colon	G1T3N0M0	ND	Failure	ND

HROC57	43/m	Right colon	G3T3N2M1	NA^§^	Success	ND

HROC59	76/m	Right colon	G2T3N1M1	spStd	Success	ND

HROC60	71/m	Right colon	G2T2N0M0	CIMP-H	Success	ND

HROC61	57/m	Rektum	G3T3N0M0	spStd	ND	Failure

HROC62	84/f	Right colon	G3T4N2M0	spStd	ND	Success

HROC63	81/f	Left colon	G2T4N0M0	spStd	Success	Failure

HROC64	71/m	Left colon	G2T2N0M0	spStd	ND	Success

HROC65	73/f	Right colon	G3T3N2M1	spStd	Success	Success

HROC66	75/m	Rektum	G2T3N2M0	spStd	ND	Failure

HROC67	54/m	Left colon	G2T3N1M0	spStd	ND	Failure

HROC68	84/m	Left colon	G2T4N2M0	spStd	Success	Success

HROC69	62/m	Right colon	G3T3N0M1	spStd	Success	Failure

HROC70	65/f	Right colon	G3T4N1M0	spStd	Success	Success

HROC71	52/m	Right colon	G2T3N0M0	HNPCC	ND	Success

HROC72	61/m	Right colon	G2T3N2M1		ND	Success

HORC73	69/m	Left colon	G2T3N0M0		ND	Failure

HROC74	80/m	Left colon	G2T4N0M0	spStd	ND	Success

HROC75	58/m	Left colon	G2T3N0M0	spStd	ND	Success

HROC78	75/m	Right colon	G3T3N0M0	CIMP-H	ND	Success

HROC80	72/m	Right colon	G2T3N2M1		ND	Success

HROC81	21/f	Right colon	G3T4N0M1	spStd	ND	Success

HROC82	62/m	Right colon	G2T3N0M0		ND	Success

HROC83	85/f	Right colon	G2T3N1M1		ND	Failure

HROC84	88/f	Left colon	G2T3N0M0	spStd	ND	Success

HROC85	65/m	Rektum	G2T3N0M0	spStd	Success	ND

HROC86	79/f	Left colon	G2T3N1M0	spStd	Success	ND

HROC87	76/f	Left colon	G3T3N0M0	spMSI	ND	Success

HROC88	69/f	Right colon	G2T3N1M0		ND	Success

HROC89	80/m	Rektum	G2T3N0M1	spStd	Success	Success

* Right colon (coecum to splenic flexure), left colon (descending colon and sigmoid), Rektum. † Abbreviations for molecular types of colorectal carcinomas. ‡ ND - not determined/not done. § NA - not applicable.

Xenografting of 23 tumors fresh from surgery was successful in 17 cases (74% take). By comparison, 31 tumors were xenografted after cryopreservation for 6 to 643 days, and this was successful in 22 cases (71% take - summarized in Table 2), Thus, there was not observed a statistically significant difference in the overall success rates between fresh (n = 17/23) and cryopreserved (n = 22/31) samples (p = 0.815, Fisher's exact T-test). As most animals received bilateral xenografts comparison between total numbers of fresh and cryopreserved xenografts could be made. Again, the difference was not statistically significant although a trend to reduced success after cryopreservation was observed (n = 34/68; 50% fresh, n = 40/130; 31% cryopreserved, p = 0,098; Table 1). In two cases, where xenografts did not grow successfully after cryopreservation, enough frozen tumor pieces were left to repeat xenografting at a much later date (643 and 489 days), this time with success (animals M3 and M4 of HROC48 and HROC55; Table 1, underlined).

For 6 of the 48 tumors, sufficient material was available to attempt both xenografting of tumor tissue fresh from surgery and xenografting of tumor tissues after cryopreservation (13 to 103 days). This was successful for all the tumor tissues xenografted fresh from surgery, and for 4 cases of cryopreserved tissues (67%; Table 2). Details on the xenograftings and their outcomes are given in Table 1.

Re-transplantation of xenograft tumors after cryopreservation was attempted for 11 tumors, and this could be carried out successfully in all cases. These tumor tissues were from passages 2 to 8 and had been cryopreserved for 56 to 455 days (Table 3). In terms of grafts, 47 of 54 grafts were accepted (87%; Table 3).

**Table 3:** Successful re-xenografting after cryopreservation.

Tumor-ID	Passage number	Days frozen	Success rate by grafts
HROC24	3	148	2/2

HROC29	6-8	147, 182, 189	7/10

HROC32	5	168	4/4

HROC39	2	164	4/4

HROC40	2	191	4/4

HROC46	2	455	7/8

HROC50	2, 3	120, 168	5/6

HROC53	2, 3	84, 168	6/6

HROC54	2	56	2/2

HROC59	2	119	2/2

HROC71	2	141	4/4

**Figure 1:**
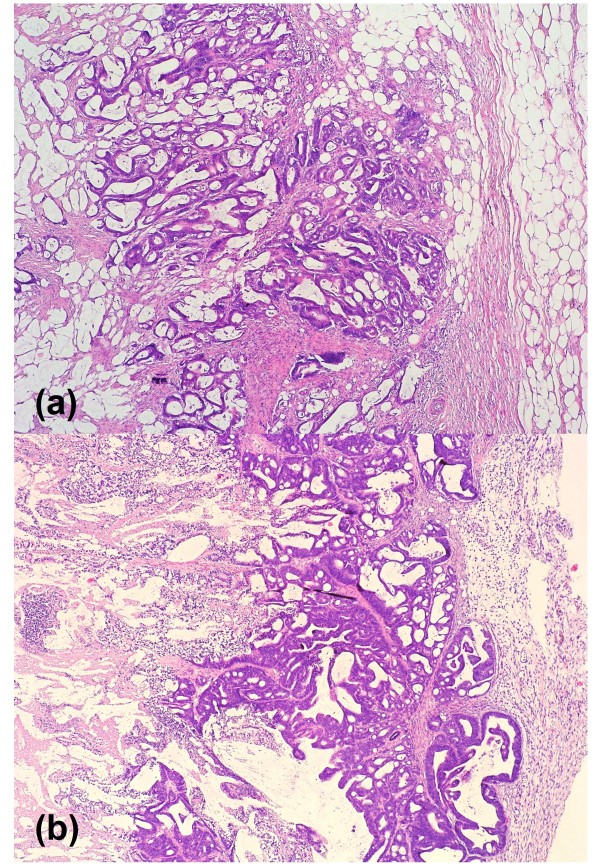
**Tumor morphology**. Examples of (a) primary tumor (HROC50) and (b) its xenograft. Compared to its primary, the tumor architecture, growth pattern, and cytological features are well preserved in the xenograft tumor.

As expected, histologically the xenograft tumors closely resembled their primaries (Figure 1). PCR studies amplifying part of the human mitochondrial cytochrome b gene gave further proof of the human origin of these tumors (data not shown). Furthermore, the molecular analyses revealed that xenografting could be carried out successfully for all the molecular types of CRC without any evident bias. Specifically, xenografts were obtained for 27 spStdCRC, 6 spMSI-H tumors, 2 HNPCC-type tumors, and 3 CIMP tumors.

Generally, xenografting was well tolerated by the animals. No signs of superinfection or suppuration were seen in any of the cases and consequently, no death from infection occurred. This was even true for those cases where tumor samples contained bacteria as judged from infections observed in parallel cultures in vitro (data not shown). In this series, we never observed metastases.

## Discussion

In this study, we were able to show that tumor tissue from primary CRC surgical resection specimens can be cryopreserved and successfully xenografted into mice even after prolonged periods of storage (up to 643 days). Even though take rates with this procedure seem to be lower than with tumor tissue used fresh from surgery (71% overall take for cryopreserved vs. 74% overall take for fresh tissues), this difference was not of statistical significance. Besides, it is still satisfactory and compares favourably to xenograft take rates reported by other groups [12-15]. Not unlikely, take rates could even be improved by increasing the number of implantations or by Matrigel-soaking of tumor pieces before xenografting as suggested by Fujii et al. as well as by Sorio et al. [14,15]. The latter group was also the first to report a successful cryopreservation technique for pancreatic cancers [14]. As a technically very simple method, cryopreservation of CRC tumor tissue prior to xenografting as reported here may be quite appealing to both clinical and basic researchers alike for the following reasons:

(1) Cryopreservation before xenografting considerably reduces logistic constraints. If tumor tissue is xenografted fresh from surgery the work-flow is very complex. At first liaison will have to be made with the surgical colleagues for the resection specimen to be brought to the pathology department. From there the tumor tissue is handed over to the xenograft-team, often past usual work-hours; and presuming in any case that there are animals ready for use in the breeding-facilities on the day of surgery. Obviously, putting a break into this will make the procedure much easier.

(2) Cryopreservation before xenografting facilitates collection of large numbers of tumor specimens, even from different centres. This may be important for pre-clinical studies addressing e.g. the effects of targeted therapies as it has been shown that human tumor xenografts can be used for prediction, especially when panels are used [16,7-9]. Conceivably, even, cryopreservation could allow post hoc analysis and propagation in xenografts of those patients' tumor tissue that have responded (or not responded) to specific therapies.

(3) Cryopreservation before xenografting allows preselection of frozen tumor samples out of a larger collection. This drastically reduces time and resources needed to obtain individual xenografts of a desired molecular type. Important to note in this respect is, apparantly by our procedure none of the major molecular types of CRC would be excluded.

(4) Finally, we observed that cryopreservation of established xenografts of CRC samples for later re-transplantation is particularly successful (100% take in terms of cases and 87% in terms of grafts). This may be interesting if low-passage xenografts are desired, as it has been shown that similar to cell lines xenografts may acquire additional mutations and changes in the karyotype [17,12]; and it is very convenient in relieving laboratories from the necessity of continuous passaging. Moreover, it will greatly facilitate the exchange of well-characterized models between different institutions.

## Conclusions

In the presented study we demonstrate the feasibility of xenografting of clinical CRC specimens after a transient cryopreservation step. A statistical comparison of the success rates with and without cryopreservation revealed no significant difference. In addition, we show that cryopreservation of established xenograft tumors for re-xenografting is particularly feasible. And finally, we show that a balanced distribution of the different molecular classes of colorectal carcinomas will be obtained using this procedure. These findings may have immediate impact on the improvement of preclinical drug testing procedures. To the best of our knowledge; this has never been done for CRC.

## Abbreviations

CIMP-H: CpG island methylator phenotype; CRC: colorectal carcinoma; HNPCC-type: hereditary non-polyposis-type CRC; spMSI-H: sporadic high-degree microsatellite instable CRC; spStdCRC: sporadic standard type CRC

## Competing interests

The authors declare that they have no competing interests.

## Authors' contributions

ML conceived of the study, participated in its design and coordination, data analysis, and writing of the manuscript. CM performed the statistics and helped to draft the manuscript. CO carried out most of the molecular analyses. UK helped to analyze and interpret the data. MK performed the human-specific PCR. EK reviewed the manuscript. FP performed the morphological analyses and participated in the interpretation of the data, and the writing of the manuscript. All authors read and approved the final manuscript.

## Pre-publication history

The pre-publication history for this paper can be accessed here:

http://www.biomedcentral.com/1471-2407/10/362/prepub
